# Resource use and clinical outcomes in patients with atrial fibrillation with ablation versus antiarrhythmic drug treatment

**DOI:** 10.1186/s12872-018-0946-6

**Published:** 2018-11-07

**Authors:** Julian W. E. Jarman, Wajid Hussain, Tom Wong, Vias Markides, Jamie March, Laura Goldstein, Ray Liao, Iftekhar Kalsekar, Abhishek Chitnis, Rahul Khanna

**Affiliations:** 1 Heart Rhythm Centre, NIHR Cardiovascular Research Unit, The Royal Brompton Hospital, and National Heart and Lung Institute, Imperial College, London, UK; 2 Franchise Health Economics and Market Access, Johnson & Johnson, Irvine, CA USA; 3 Janssen R&D US, Raritan, NJ USA; 4 Medical Device Epidemiology, Johnson and Johnson, 410 George Street, New Brunswick, NJ 08901 USA

**Keywords:** Atrial fibrillation, Catheter ablation, Anti-arrhythmic drugs

## Abstract

**Background:**

The objective of our study was to compare resource use and clinical outcomes among atrial fibrillation (AF) patients who underwent catheter ablation versus antiarrhythmic drug (AAD) treatment.

**Methods:**

A retrospective cohort design using the Clinical Practice Research Data-Hospital Episode Statistics linkage data from England (2008–2013) was used. Patients undergoing catheter ablation treatment for AF were indexed to the date of first procedure. AAD patients with at least two different AAD drugs were indexed to the first fill of the second AAD. Patients were matched using 1:1 propensity matching. Primary endpoints including inpatient and outpatient visits were compared between ablation and AAD cohorts in the 4 months-1 year period after index. Secondary endpoints including heart failure, stroke, cardioversion, mortality, and a composite outcome were compared for the 4 months-3 years post-index period in the two groups. Cox-proportional hazards models were estimated for clinical outcomes comparison.

**Results:**

A total of 558 patients were matched in the two groups for resource utilization comparison. The average number of cardiovascular (CV)-related outpatient visits in the 4–12 months post-index period were significantly lower in the ablation group versus the AAD group (1.76 vs 3.57, *p* < .0001). There was no significant difference in all-cause and CV-related inpatient visits and all-cause outpatient visits among the two groups. For secondary endpoints comparison, 615 matched patients in each group emerged. Ablation patients had 38% lower risk of heart failure (hazard ratio [HR] 0.62, *p* = 0.0318), 50% lower risk of mortality (HR 0.50, *p* = 0.0082), and 43% lower risk of experiencing a composite outcome (HR 0.57, *p* = 0.0009) as compared to AAD treatment cohort.

**Conclusion:**

AF ablation was associated with significantly lower CV-related outpatient visits, and lower risk of heart failure and mortality versus AAD therapy.

## Background

Atrial fibrillation (AF) affects approximately 2% of the population and is a significant risk factor for stroke and heart failure [1–3]. Recent estimates suggest that AF prevalence is increasingly on a yearly basis in the United Kingdom (UK), and the number of patients with AF is expected to increase from 700,000 in 2010 to as high as 1.8 million by 2060 [4]. Besides causing significant morbidity, AF is associated with considerable healthcare utilization and economic burden. In the UK, the direct costs are estimated to be as high as £244 million (2004) of which hospitalizations and prescription drugs account for 70% of the expenditure [5]. In Europe, AF has a substantial economic burden, ranging from €660 million to €3286 million; direct costs comprise up to 80% of costs [6, 7].

Treatments for AF include both pharmaceutical and non-pharmaceutical options; however, a large proportion of patients are left untreated [3, 8, 9]. Undertreatment is the result of multiple factors, including improper assessment, over-estimation of the risk of bleeding and underestimation of the risk of stroke. Clinical trials demonstrate that AF ablation supports sinus rhythm more effectively than antiarrhythmic drugs (AAD) in patients with symptomatic, paroxysmal AF [10, 11]. Ablation treatment has been shown to be cost-effective as compared to AAD treatment for AF [12], and is associated with improvement in patient-reported health-related quality of life [13]. In addition, retrospective cohort studies using large databases have found significantly lower rate of stroke and other adverse outcomes associated with AF ablation as compared to other treatment alternatives including AAD drugs [14, 17]. In one such study, Jarman et al. (2017) found significantly lower rates of stroke among AF patients undergoing ablation procedure as compared to AF patients who did not have an ablation or had cardioversion [15].

The current study builds on earlier clinical and observational research on understanding the difference between ablation and AAD treatment for AF. The primary objective of the study was to compare health care resource use over a 1-year period among patients with AF who underwent catheter ablation as compared to AAD treatment. Secondary objectives included comparison of stroke/transient ischemic attack (TIA), heart failure, direct current cardioversion (DCCV), death, and a composite of these outcomes among AF patients with ablation versus AAD treatment.

## Methods

### Data source(s)

The UK Clinical Practice Research Datalink (CPRD), a longitudinal database of more than 11 million patients representing 7% of the total UK population [18], was used for the current study. CPRD data has been utilized in over 1800 publications including drug safety, practice guidelines and clinical guidelines (www.cprd.com). Along with CPRD, linkage with Hospital Episode Statistics (HES) was performed to identify the patients with and without ablation for AF. HES data contains detailed information on the fields from the admitted patient, outpatient, accident and emergency (A&E) and adult critical care.

### Study design

This retrospective longitudinal cohort design studied patients ≥18 years of age diagnosed with AF and treated with either ablation or AADs (specified as amiodarone, disopyramide, dronedarone, flecainide, propafenone, and sotalol) during an evaluation period from 2008 to 2013. For the ablation cohort, the earliest date of the ablation procedure was defined as the index date. Since catheter ablation is recommended only for patients that have failed to show improvement on prior AAD therapy, patients in the AAD cohort were required to have prescriptions for at least two different AAD drugs during the study period, to ensure comparisons between the two cohorts were conducted between like populations (e.g., all patients had failed or lacked sufficient improvement on first AAD). For the AAD cohort, the date of the second AAD was defined as the index date. All patients were required to have 12 months of complete medical record data prior to index date (referred to as the baseline or pre-index period), as well as 12 months of post-index data. Consistent with past approaches and treatment guidelines [11, 19], we implemented a 3-month blanking period for outcomes assessed across both groups.

Patients were excluded if any of the following criteria were met: ablation procedures performed during the 12-month pre-index period (ablation cohort) or ablation procedures performed during 12-months pre- and post-index period (AAD medication cohort); procedural code for implantation of a pacemaker or implantable cardioversion defibrillator in the 12-month pre-index period; surgical ablation performed in the 12-month pre-index period including those ablation procedures that are performed concomitantly with open heart surgery for valvular, ischemic, or congenital heart disease; valvular procedures performed in the 12-month pre-index period; and left atrial appendage occlusion procedure in the 12-month pre-index period.

### Study measures

Patient age and gender were recorded on the index date. Patient comorbidities were recorded during the baseline (pre-index) period based on the presence of specific ICD codes and included ischemic heart disease with and without myocardial infarction, heart failure, cardiomyopathy, hypertensive heart disease without heart failure, valvular heart disease, conduction system disease, Wolff-Parkinson-White syndrome, other arrhythmias, hypertension, diabetes, obstructive sleep apnea, chronic obstructive pulmonary disease (COPD), acute renal failure, stroke/TIA, DCCV, and hyperthyroidism. The Charlson comorbidity index (CCI), which is an aggregate measure of comorbidity created by using select diagnoses associated with chronic disease (e.g., heart disease, cancer), was also assessed. The CCI includes 17 medical conditions and weights these conditions from + 1 to + 6 [20, 21]. Patients’ stroke risk was measured using the CHADS_2_-VASc index with a maximum score of 9; it was calculated using the presence of congestive heart failure, hypertension, Type II Diabetes Mellitus (T2DM), stroke, age, prior MI disease. Cardiovascular-related inpatient and outpatient visits in the 12-month pre-index period were assessed. Lastly, rate-control and anticoagulant medications used in the 12-month pre-index period were also assessed.

### Outcome measures

Primary outcome measures, assessed during the post-blanking 4-month to 12-month period, were defined as the average number of all-cause and cardiovascular-related inpatient admissions and outpatient visits. Secondary outcome measures included inpatient readmissions with any recorded diagnosis of heart failure, stroke/transient ischemic attack (TIA), DCCV, death, and a composite measure of these outcomes occurring during the post-blanking 4-month to 3-year period. Patients were followed from the index date until record of event, death, or end of follow-up, whichever came first.

### Data analysis

The ablation and AAD cohorts were first matched using the propensity score matching technique, implementing a multivariate logistic regression between patients who underwent ablation and those receiving AADs to assess factors predicting the use of ablation procedure among AF patients. Factors included in matching were age and gender (recorded at index date), comorbidities at baseline, drug utilization, and baseline resource use. After propensity score matching, the average number of inpatient admissions and outpatient visits (primary outcome measures) were compared between cohorts. Comparisons between groups were performed using 2-sample t-test. As part of the secondary outcomes assessment, a separate propensity score matching was conducted, as the pre-match ablation and AAD sample involved was different than the pre-match sample for the primary objective assessment. For secondary objectives, the ablation and AAD sample were followed for a period of three years and were therefore not required to have 12-month post-index continuous enrollment (unlike primary objective assessment, where this criterion was applied). Secondary outcomes were studied in the propensity-matched sample using log-rank test. Further, regression analysis using Cox Proportional Hazards modeling was conducted to examine the relationship between treatment status and secondary outcome adjusting for any significant standardized differences emerging post-matching. All analyses were conducted using SAS for Windows and statistical significance was set a-priori at *p* < 0.05 (two-sided).

## Results

**Fig. 1:**
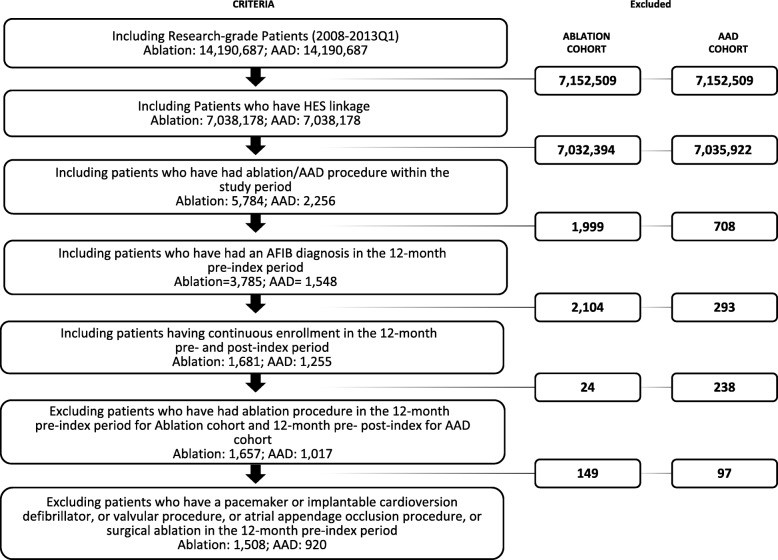
Sample attrition

A total of 1508 patients in the ablation cohort and 920 patients in the AAD cohort were included (Fig. 1). After applying propensity score matching, a total of 558 patients were included in each cohort. The post-match sample balanced well on study variables as indicated by standardized difference scores (which were less than 0.10 for all variables except index year and Wolff-Parkinson White syndrome) between the two groups (Table 1).

**Table 1:** Pre-match and post-match sample characteristics for primary outcome assessment

*Variable*	Before Propensity Score Matching			After Propensity Score Matching		
	AAD cohort (*n* = 920)	Ablation cohort (*n* = 1508)	Standardized difference	AAD cohort (*n* = 558)	Ablation cohort (*n* = 558)	Standardized difference
Age	68	62	−0.5075	65	65	−0.0535
CCI score	0.7	0.72	0.0445	0.7	0.7	0.0027
CHA_2_DS_2_-VASc score	2.11	1.63	−0.322	1.9	1.9	0.0168
Cardiovascular-related outpatient visits (pre-index)	2.99	2.98	−0.3613	1.2	1.28	0.06
Cardiovascular-related inpatient visits (pre-index)	1.38	2.12	0.5151	1.75	1.83	0.0593
Female	45.65%	28.58%	0.359	34.41%	35.30%	−0.0188
Year of index date						
2008	20.11%	13.26%		19.53%	15.05%	
2009	19.24%	15.72%		19.89%	15.41%	
2010	19.24%	19.76%		18.46%	18.10%	
2011	17.72%	17.71%		17.20%	15.05%	
2012	13.80%	18.04%		12.72%	19.18%	
2013	9.89%	15.52%	0.2698	12.19%	17.20%	0.2653^a^
DCCV	19.78%	25.73%	0.1422	24.37%	23.84%	−0.0126
Ischemic heart disease	17.07%	14.46%	−0.0716	18.28%	19.53%	0.032
Heart failure	8.37%	9.81%	0.0503	9.68%	8.60%	−0.0373
Cardiomyopathy	2.39%	4.97%	0.1374	3.58%	3.23%	−0.0198
Valvular disease	6.63%	10.81%	0.1485	8.42%	7.89%	−0.0196
Wolff Parkinson	0.11%	2.19%	0.1961	0.18%	1.08%	0.1137^a^
Other arrhythmia	10.33%	22.35%	0.3296	14.16%	15.95%	0.0501
Hypertension	36.09%	39.66%	0.0736	41.22%	42.29%	0.0218
Diabetes	9.57%	12.00%	0.0786	11.65%	11.47%	−0.0056
Obstructive sleep apnea	0.54%	2.19%	0.1421	0.90%	1.25%	0.0348
COPD	5.54%	6.76%	0.0508	6.45%	6.63%	0.0072
Renal failure	1.20%	1.06%	−0.0127	0.54%	0.72%	0.0227
Stroke/TIA	3.59%	1.92%	−0.1018	2.69%	2.15%	−0.035
Conduction system disease	14.67%	30.97%	0.3958	19.53%	20.97%	0.0357
Hyperthyroidism	0.98%	0.20%	−0.102	0.18%	0.36%	0.0346
Rate control meds	89.57%	75.13%	−0.3855	84.77%	85.84%	0.0304
Anticoagulants	60.98%	76.46%	0.3386	72.94%	68.64%	−0.0947

^a^is significant

No significant differences in the ablation and AAD cohort emerged in terms of the average number of all-cause hospitalizations (0.70 vs 0.75, *p* = 0.086), cardiovascular-related hospitalizations (0.55 vs.0.58, *p* = 0.355), and all-cause outpatient visits (7.95 vs.8.79, *p* = 0.203). However, the average number of cardiovascular-related outpatient visits were significantly lower for the ablation cohort as compared to the AAD cohort (1.76 vs.3.57, *p* < 0.0001, Table 2).

**Table 2:** Average number of visits during the post-blanking 4-month to 12-month follow-up

		AAD Cohort *N* = 558	Ablation Cohort *N* = 558	*p*-value
All-cause inpatient visits	Mean	0.75	0.70	0.0859
	Std	1.80	1.25	
Cardiovascular-related inpatient visits	Mean	0.58	0.55	0.3547
	Std	1.38	0.99	
All-cause outpatient visits	Mean	8.79	7.95	0.2029
	Std	7.41	6.45	
Cardiovascular-related outpatient visits	Mean	3.57	1.76	<.0001
	Std	5.09	3.83	

For the secondary outcomes assessment, where patients were followed for a period of three years, a total of 1528 patients in the ablation cohort and 927 patients in the AAD cohort emerged as part of the pre-match sample. Significant standardized differences in the pre-match study cohorts were observed. After propensity matching, a total of 615 matched patients were included in each cohort, and were analyzed with respect to the secondary outcomes. The two cohorts matched well in terms of standardized differences (Table 3), with only index year emerging as significant post-matching, which was adjusted in a Cox regression model.

**Table 3:** Pre-match and post-match sample characteristics for secondary outcome assessment

*Variable*	Before Propensity Score Matching			After Propensity Score Matching		
	AAD cohort (*n* = 927)	Ablation cohort (*n* = 1528)	Standardized difference	AAD cohort (*n* = 615)	Ablation cohort (*n* = 615)	Standardized difference
Age	68	62	−0.5428	65	66	0.0613
CCI score	0.7	0.71	0.0255	0.74	0.75	0.0205
CHA_2_DS_2_-VASc score	2.11	1.63	−0.3626	1.24	1.23	0.0517
Cardiovascular-related outpatient visits (pre-index)	1.46	0.96	−0.3568	1.23	1.17	−0.0449
Cardiovascular-related inpatient visits (pre-index)	1.38	2.13	0.4898	1.76	1.80	0.0349
Female	45.63%	28.86%	0.3747	34.15%	36.26%	−0.0443
Year of index date						
2008	20.28%	13.29%		18.05%	13.98%	
2009	18.99%	15.58%		19.02%	16.26%	
2010	19.20%	19.90%		19.35%	19.19%	
2011	17.80%	17.67%		16.59%	15.45%	
2012	13.81%	18.06%		14.47%	18.05%	
2013	9.92%	15.51%	0.2612	12.52%	17.07%	0.1942^a^
DCCV	19.31%	25.59%	0.1473	25.37%	26.18%	0.0186
Ischemic heart disease	17.04%	14.53%	−0.0852	16.42%	17.56%	0.0303
Heart failure	8.41%	9.75%	0.0363	9.59%	9.92%	0.011
Cardiomyopathy	2.37%	4.91%	0.1332	3.25%	2.93%	−0.0188
Valvular disease	6.69%	10.73%	0.138	8.94%	9.11%	0.0057
Wolff Parkinson	0.11%	2.23%	0.1951	0.16%	0.65%	0.0767
Other arryhthmias	10.46%	22.58%	0.3094	14.80%	16.26%	0.0404
Hypertension	36.14%	39.66%	0.0308	41.30%	39.67%	−0.0331
Diabetes	9.39%	11.98%	0.0611	11.54%	11.71%	0.0051
Obstructive sleep apnea	0.54%	2.23%	0.1268	1.14%	1.46%	0.0287
COPD	5.50%	6.68%	0.0572	6.99%	6.34%	−0.0261
Renal failure	1.19%	1.05%	−0.0397	0.81%	0.65%	−0.0191
Stroke/TIA	3.56%	1.90%	−0.103	2.93%	1.95%	−0.0633
Conduction system disease	14.89%	31.02%	0.3682	21.14%	22.28%	0.0276
Hyperthyroidism	1.08%	0.20%	−0.0968	0.16%	0.16%	0
Rate control meds	89.64%	75.13%	−0.3948	84.88%	84.72%	−0.0045
Anticoagulants	60.95%	76.46%	0.3266	72.68%	70.57%	−0.0469

^a^is significant

**Fig. 2:**
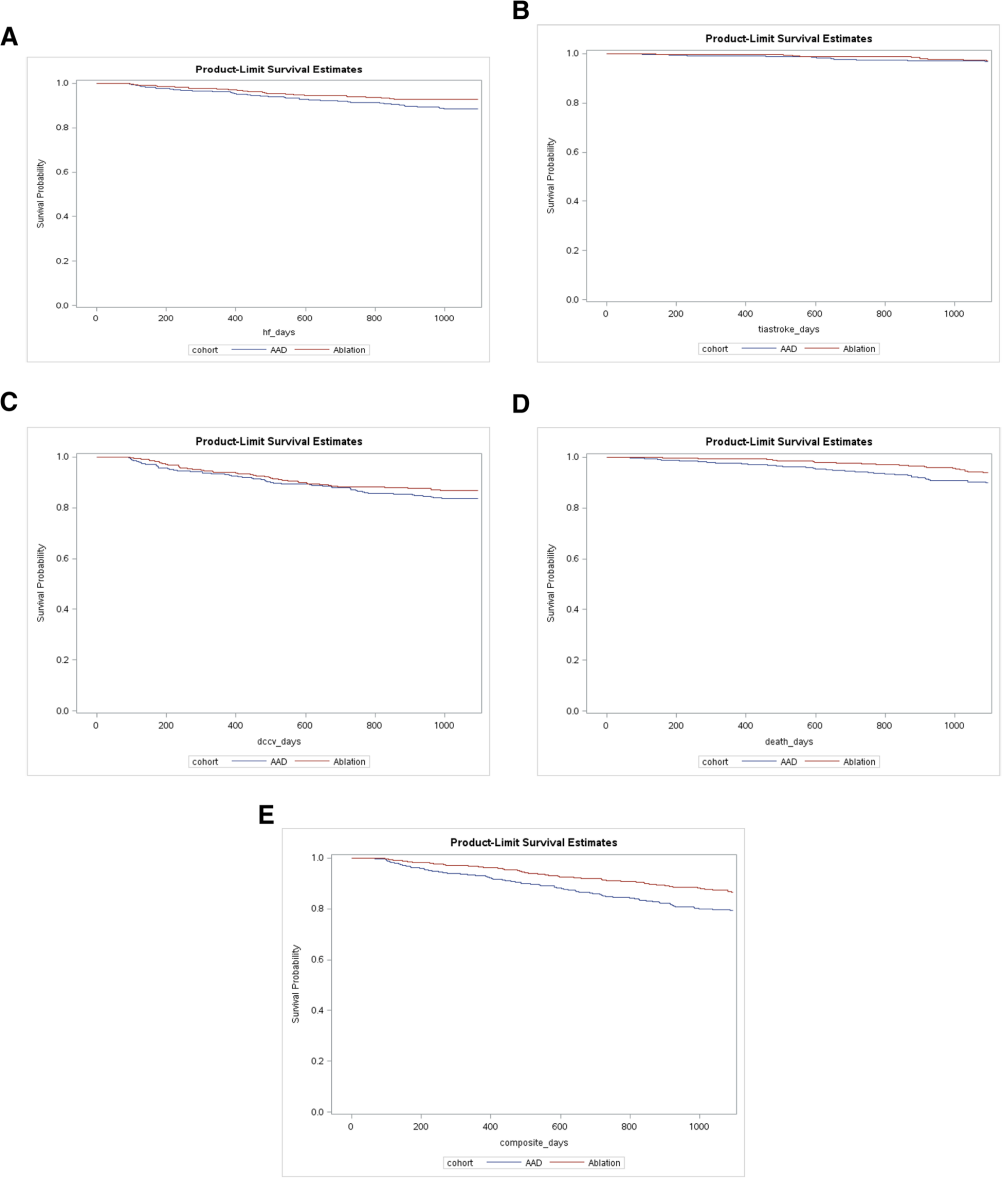
Survival probability for ablation and AAD cohort in the post-index 4-month to 3-year time-period for secondary outcomes **a** Heart failure. **b** Stroke/TIA. **c** DCCV. **d** Death. **e** Composite outcome (including heart failure, stroke/TIA, DCCV, death)

Figure 2 (a-e) depicts the survival curves for the ablation and AAD cohort for secondary outcomes. Results from comparing survival curves for heart failure were significant, with the ablation cohort having lower likelihood of heart failure over the three-year period as compared to the AAD cohort (*p* = 0.0342 for the log-rank test; Fig. 2a). No significant differences in survival curves for stroke/TIA (*p* = 0.579 for log-rank test; Fig. 2b) or DCCV (*p* = 0.2018 for log-rank test; Fig. 2c) emerged between the two cohorts. The ablation cohort was found to have a significantly lower rate of death (*p* = 0.0112 for log-rank test; Fig. 2d) and of the composite outcome (*p* = 0.0012 for log-rank test; Fig. 2e) as compared to AAD cohort.

Results from the regression analysis revealed that patients in the ablation cohort had a 38% lower rate of heart failure (Hazard Ratio [HR]: 0.624; *p* = 0.0318) as compared to the AAD cohort. Patients in the ablation cohort had ~ 50% lower mortality rate as compared to the AAD cohort (HR: 0.507, *p* = 0.008). The ablation cohort was ~ 43% less likely to incur any defined events (composite outcome) during the three-year follow-up as compared to the AAD cohort (HR: 0.578; *p* = 0.001). No significant difference in likelihood of stroke/TIA (HR: 0.82; *p* = 0.623) and DCCV (HR:0.793; *p* = 0.169) were observed between the ablation and AAD cohort.

## Discussion

Current guidelines for AF management recommend AADs as the first-line therapy [11, 19, 22–25]. The 12-month AF recurrence rate for patients treated with AADs ranges from 24 to 63% [11, 22, 25]. Among drug refractory AF patients, catheter ablation is the recommended treatment option [19]. A meta-analysis of nine studies found significantly better success rate for AF treatment with catheter ablation both in the short-term (< 1 year) [OR, 10.84; 95% CI, 5.83–20.16; *P* < 0.001] and long-term (> 1 year) [OR, 7.65; 95% CI, 1.97–29.73; *P* = 0.03] as compared to AADs [26]. While existing randomized controlled trial (RCT) experience demonstrates superior efficacy in terms of reduction of AF recurrence and symptoms with ablation over AADs [11, 22, 23, 25], it is important to explore the outcome variation in other parameters such as resource utilization and clinical events/hospitalizations among the two treatments in a real-world environment.

Using one of the largest nationally representative databases in the UK, our study provided insights into a short- and long-term outcomes comparison between ablation and AAD treatment among patients with AF. When assessing the cost-effectiveness of ablation treatment as compared to AAD treatment among AF patients, Rizzo et al. (2012) found an incremental cost-effectiveness ratio of £12,500 to £15,300 per quality-adjusted life-year for the ablation cohort as compared to the AAD cohort (QALY) [12]. Further, the authors reported the quality-adjusted life expectancy to be between 11.75 to 12.20 years for catheter ablation and 11.00 to 11.35 years for AAD cohort [12]. We conducted propensity score matching to minimize heterogeneity between the groups and normalize key influential factors including demographics, and underlying comorbidity status. Our study adds to the existing evidence highlighting the clinical outcome and resource utilization benefit associated with ablation as compared to AADs among AF patients.

Consistent with past studies, our study indicates lower resource use and better outcomes associated with ablation treatment as compared to AAD treatment among patients with AF. In the 12-month period post-index treatment, AF patients treated with AADs had more than twice the average number of cardiovascular-related outpatient visits as compared to those treated with ablation (3.57 [SD: 5.09] vs. 1.76 [SD: 3.83], *p* < 0.0001). When assessing outcomes over a longer term (3-year period), AF patients treated with ablation procedure were found to have ~ 38% lower likelihood of heart failure, ~ 50% lower likelihood of death, and ~ 43% lower likelihood of a composite outcome (including heart failure, stroke/TIA, DCCV, death) as compared to those treated with AADs. These results are consistent with earlier observational evidence supporting ablation procedure. In a recent observational study, Mansour et al. (2018) found 41% greater likelihood of thromboembolic event and 13% greater likelihood of cardiovascular hospitalization among AF patients undergoing AAD therapy as compared to ablation treatment [16]. Besides resource use and clinical outcome benefit, the significant reduction in mortality observed among AF patients treated with ablation in our study highlights the potential health benefit accrual associated with ablation as compared to AAD therapy. Similar to our study, Jarman et al. (2017) observed lower likelihood of morality among AF patients undergoing ablation procedure as compared to AF patients who did not had ablation or had cardioversion [14]. As healthcare resources become scarce, treatment approaches including ablation for AF could offer payers significant economic benefits as compared to conventional drug treatment.

### Study limitations

As with all observational studies, our study also has some limitations. Considering that we used a secondary healthcare database for this study, coding errors and misclassifications could have influenced results. Under-reported or missing diagnoses, based on patient’s choice (not to seek care) or access challenges may also exist, though the extent of such occurrences may be minimal. The study did not assess patient quality of life differences between the two cohorts associated with long term use of AADs or ablation because these measures are not routinely captured in healthcare databases. The database used in the study did not have any procedure-related details. For example, we were unable to examine the catheter technology, procedure time, and fluoroscopy time. As ablation catheter technology has evolved, there are likely to be variation in success rate within ablation catheters, with newer catheters having improved outcomes as compared to earlier generations. For instance, the contact force-sensing radiofrequency ablation catheters are shown to lead to 37% decrease in AF recurrence over a 12-month follow-up period when compared to radiofrequency catheters without contact force-sensing technology [27]. Future studies that have technology information available could examine the variation in outcomes between AADs and newer ablation catheters. Lastly, one of the main limitations of observational studies like ours is the lack of randomization. Unlike clinical trials, where randomization could be used to alleviate selection bias, observational studies like ours must rely on other techniques to reduce such bias. Though we achieved good balance among the two groups for primary and secondary outcome comparison using the propensity score matching technique, unmeasured confounders could have existed and influenced the results.

## Conclusions

This study builds on existing literature highlighting the significant reduction in resource utilization as well as improvement in morbidity and mortality outcomes associated with ablation as compared to AAD treatment for AF. For patients, payers, and providers, the incremental benefits indicate ablation to be a valuable treatment approach for AF as compared to drug therapy. For patients, the morbidity and mortality benefit associated with ablation are likely to translate to clinical benefits over AAD therapy. For providers, ablation offers a useful approach to treat AF with improved clinical outcomes, and for payers, ablation is likely to lead to sustained economic savings as compared to AAD therapy.

## Abbreviations

AAD: Antiarrhythmic drugs
AF: Atrial fibrillation
CPRD: Clinical Practice Research Datalink
DCCV: Direct Current Cardioversion
HES: Hospital Episode Statistics
TIA: Transient Ischemic Attack
UK: United Kingdom (UK)
